# Correlation Between Radiographic and MRI Posterior Tibial Slope Measurement on a Pediatric Population

**DOI:** 10.3390/jcm15010064

**Published:** 2025-12-22

**Authors:** Clémence Peufly, Lyes Chaal, Elie Chouffani, Romir Patel, Sebastien Pesenti, Matthieu Ollivier, Antoine Piercecchi

**Affiliations:** 1 Department of Orthopedic Surgery, Institute for Locomotion, Aix-Marseille University, 13284 Marseille, Franceolliviermt@gmail.com (M.O.); 2 Department of Biomechanics, Institute for Locomotion, St Marguerite Hospital, APHM, CNRS, ISM, Aix-Marseille University, 13284 Marseille, France; 3 Department of Orthopaedic Surgery, Massachusetts General Hospital, Boston, MA 02114, USA

**Keywords:** pediatric, posterior tibial slope, MRI measurement, ACL injury, ACL reconstruction

## Abstract

**Background/Objectives:** Posterior tibial slope (PTS) is an established risk factor for anterior cruciate ligament (ACL) injury in adults. In pediatric population, this relation is less established, and the PTS measurement is not clearly defined. To determine the agreement between X-ray (XR) and magnetic resonance imaging (MRI) PTS measurements and to establish an MRI cutoff corresponding to the standard radiographic ≥12° definition of “high slope”. **Methods:** In this retrospective study, 108 adolescent knees with ACL rupture underwent paired XR and MRI evaluation by two reviewers. Agreement was assessed with Pearson and Spearman correlation, intraclass correlation coefficient (ICC), Bland–Altman analysis, and Deming regression. Diagnostic performance of MRI thresholds was compared with XR ≥ 12° as reference. **Results:** Mean PTS was higher on XR (10.2 ± 3.1°) than on MRI (8.4 ± 2.8°), with a systematic bias of +1.8° revealed by Bland–Altman analysis. These two measurements showed strong positive correlation (r = 0.602, *p* < 0.001) and moderate concordance (ICC = 0.506, 95% CI, 0.186–0.696, *p* = 0.0015). Individual differences ranged up to ±5° between modalities. Using XR ≥ 12° as reference for “high slope,” ROC analysis identified an optimal MRI cutoff of 8.8° with excellent diagnostic accuracy (AUC = 0.841, 95% CI, 0.760–0.922). **Conclusions:** Radiographic measurements systematically overestimate PTS relative to MRI. Numeric thresholds are not interchangeable between modalities. An MRI cutoff of approximately 9° corresponds to the radiographic ≥12° definition of high slope and may serve as a pragmatic reference for interpreting MRI-based measurements in pediatric patients, requiring further validation.

## 1. Introduction

Posterior tibial slope (PTS) is an important biomechanical factor contributing to knee joint stability [1]. Several studies have identified it as a key anatomical determinant influencing the risk of anterior cruciate ligament (ACL) injury and graft failure after ACL reconstruction in adults [2,3,4]. A steeper PTS has been associated with increased anterior tibial translation and greater mechanical stress on the ACL and subsequent ACL graft [4].

PTS is historically measured on lateral radiographs (XR), with a threshold of ≥12° frequently used to define a “high slope” in adults [5]. Several measurement techniques have been described using different anatomical landmarks such as the proximal tibial surface, the anterior or posterior cortical slope, and the anatomic or mechanical axis [6]. Alternative measurement methods have also been proposed using magnetic resonance imaging (MRI) or computed tomography (CT) [7]. All these methods show significant variability in the values obtained, and to our knowledge, no standardized cutoff currently exists for cross-sectional imaging modalities [8,9,10].

The relationship between PTS and ACL injury has been only sparsely investigated in pediatric populations, with a limited number of studies [11,12,13,14,15,16] using either radiographic or MRI-based measurements. The variability and discrepancies observed between these methods underline the need to clarify their correlation and to define an MRI threshold equivalent to the conventional radiographic definition of a “high slope” [12,17].

This study aims to compare and determine the agreement of PTS measurements between radiographic and MRI assessments in a pediatric population with ACL injuries, and to identify an MRI-based cutoff corresponding to the standard radiographic ≥12° definition used in adults. We hypothesized that radiographic and MRI measurements would show strong correlation but systematic differences, preventing direct interchangeability between modalities.

The null hypothesis was that radiographic and MRI measurements of medial posterior tibial slope show no systematic bias and are interchangeable for clinical assessment.

## 2. Materials and Methods

### 2.1. Study Design and Population

The study was designed as a retrospective study of patients’ imaging. All patients younger than 18 years who underwent primary isolated ACL reconstruction (ACLR) with autograft between January 2018 and April 2025 were screened for eligibility. Skeletal immaturity was defined as chronologic age < 18 years, in accordance with institutional practice.

According to national regulations, institutional review board approval was not required for this retrospective observational study based on anonymized data.

### 2.2. Measurement Methods

Medial tibial slopes were measured on both lateral radiographs and MRI scans. For the radiographic protocol, the medial posterior tibial slope (MPTS) was measured on standardized lateral leg radiographs obtained in full extension, using the validated circle-fit technique [18].

**Figure 1:**
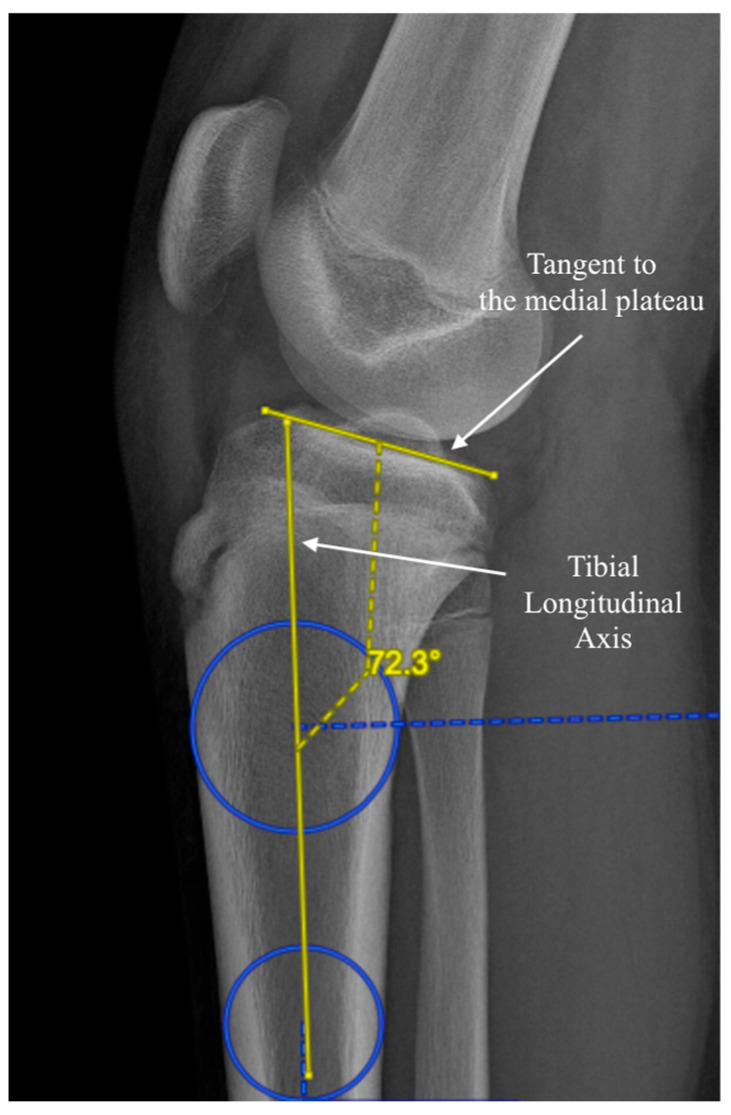
Lateral Knee Radiograph demonstrating the 2-circle method in measuring the medial posterior tibial slope (MPTS). The tibial anatomical axis is defined by two circles placed along the tibial shaft. The posterior tibial slope is calculated as 90° minus the angle (α) between this axis and the tangent to the medial tibial plateau.

The proximal tibial axis was established by fitting two circles along the tibial shaft—one immediately distal to the tibial tuberosity and another placed at least 5 cm farther down the diaphysis. The posterior tibial slope was then calculated as 90° minus the angle formed between this anatomical axis and a tangent drawn across the anterior and posterior cortices of the medial tibial plateau, as illustrated in Figure 1.

Radiograph quality was assessed according to three criteria: posterior femoral condyle overlap < 5 mm, distal condyle overlap < 5 mm, and a minimum 5 cm distance between the proximal and distal axis points. In adults, a tibial slope ≥ 12° is considered a risk factor for ACL injury; this threshold was therefore used in the present study as a reference value [5].

**Figure 2:**
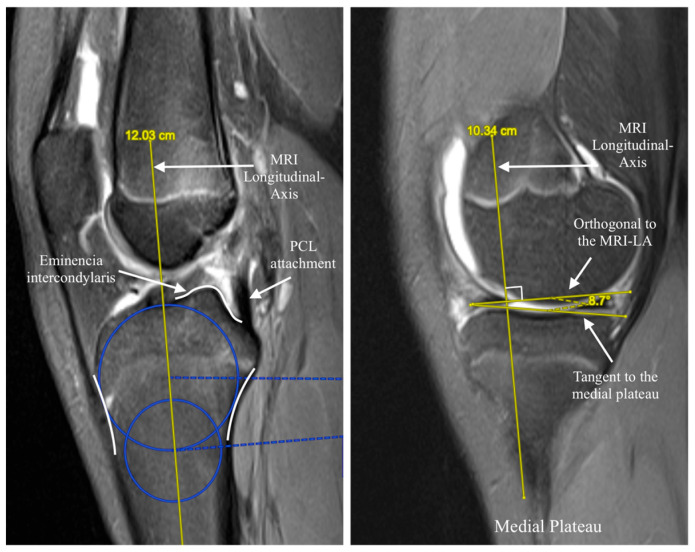
Sagittal MRI slice of the knee demonstrating the 2-circle method for measuring the medial posterior tibial slope (MPTS). (**Left**): Defining the MRI Longitudinal Axis of the tibia (MRI-LA). (**Right**): Measuring the angle between the tangent to the medial plateau and the line orthogonal to the MRI-Longitudinal Axis.

For the MRI protocol, non-contrast MRI scans (1.5 T, MAGNETOM Aera, Siemens Healthineers, Erlangen, Germany) with sagittal T1-weighted 3 mm slices were analyzed. The medial tibial slope was measured using the validated Hudek circle method [19], which first reconstructs the tibial axis on central sagittal images before calculating the slope on the central slice of the medial compartment, as shown in Figure 2. These techniques were selected because they are among the most widely validated and reproducible methods for assessing medial posterior tibial slope on radiographs and MRI, respectively, and allow comparison with prior literature.

All measurements were performed independently by two orthopedic surgeons with experience in knee imaging interpretation. Each observer performed measurements independently and was blinded to the results of the other imaging modality at the time of assessment. Measurements were performed once per observer, and no duplicate measurements were obtained; therefore, inter-observer reliability statistics could not be calculated. Image analysis was conducted using Centricity Picture Archiving and Communication System (PACS, GE Healthcare, Chicago, IL, USA).

### 2.3. Statistical Analysis

A correlation matrix analysis was performed to assess the relationship between the two measurement methods using both Pearson’s and Spearman’s correlation coefficients. Correlation values between 0.40 and 0.59 were defined as “moderate positive correlation”, between 0.60 and 0.79 as “strong positive correlation”, and between 0.80 and 1.00 as “very strong positive correlations”. Intraclass correlation coefficients (ICC) were calculated for each of the measurement methods to evaluate interrater reliability. Deming regression (λ = 1) was used to evaluate the relationship between the two measurements and estimate systematic bias. Bland–Altman plots were generated to assess agreement between the different measurement methods and to identify any systematic difference between the methods. Diagnostic agreement was evaluated by receiver operating characteristic (ROC) curve analysis using XR ≥ 12° as reference. The optimal MRI cutoff was determined by maximizing Youden’s index. Diagnostic indices included sensitivity, specificity, positive predictive value (PPV), negative predictive value (NPV), accuracy, and Cohen’s κ. McNemar’s test was used to assess systematic classification bias. Subgroup analyses assessed differences by sex using independent *t*-tests, and correlations with age, Body Mass Index (BMI), and weight were evaluated using Pearson correlation coefficients. A *p*-value of less than 0.05 was considered statistically significant. All statistical analyses were conducted using R version 4.5.1 (R Foundation for Statistical Computing, Vienna, Austria).

This study was conducted and reported in accordance with the GRRAS (Guidelines for Reporting Reliability and Agreement Studies), and the completed checklist is provided as Supplementary Material (File S1).

## 3. Results

### 3.1. Cohort Characteristics

Baseline characteristics are presented in Table 1. A total of 108 patients with both XR and MRI scans available were included for analysis. The cohort comprised 74 males (68.5%) and 34 females (31.5%), with a mean age of 13.9 ± 2.0 years, mean weight of 58.0 ± 14.1 kg, and mean BMI of 22.1 ± 4.1 kg/m^2^.

### 3.2. Primary Measurements and Correlation

The mean medial posterior tibial slope measured on XR was 10.2 ± 3.1°, and on MRI was 8.4 ± 2.8°. XR and MRI measurements showed a strong positive correlation, with a Pearson correlation coefficient of r = 0.602 (95% CI, 0.465–0.710, *p* < 0.001) and a Spearman rank correlation of ρ = 0.604 (*p* < 0.001). The intraclass correlation coefficient between modalities was 0.506 (95% CI, 0.186–0.696, *p* = 0.0015), indicating moderate concordance.

### 3.3. Agreement Analysis

**Figure 3:**
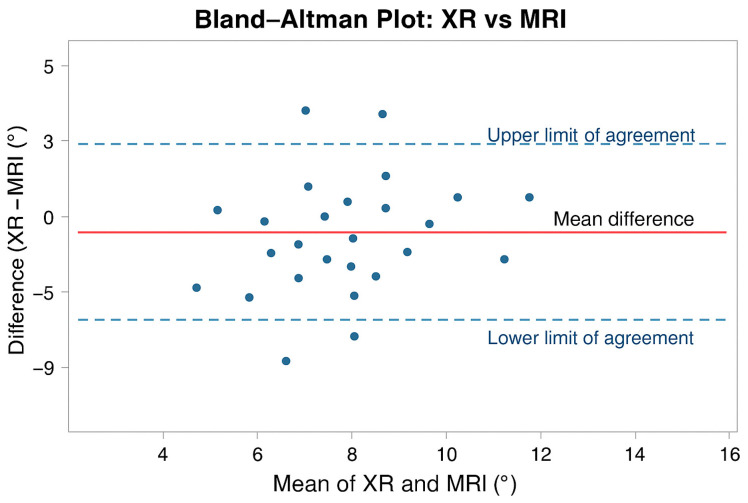
Bland–Altman plot comparing X-ray and MRI measurements of posterior tibial slope. The solid horizontal line represents the mean bias (+1.8°), and dashed lines represent the 95% limits of agreement.

As illustrated in Figure 3, the Bland–Altman plot showed substantial variability between modalities, with a mean bias of +1.8°, radiographic measurements being consistently higher than MRI. Individual differences reached approximately ±5°. Deming regression (λ = 1) produced the equation MRI = −0.180 + 0.840 × XR, confirming that MRI systematically yields lower values as XR measurements increase.

### 3.4. Diagnostic Performance

**Figure 4:**
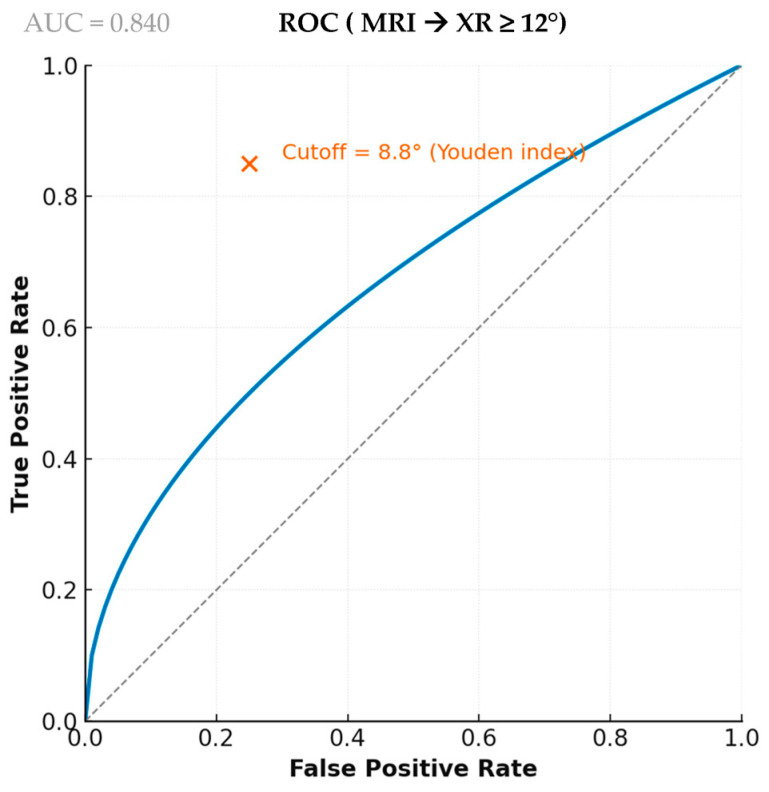
ROC Curve Illustrating the Diagnostic Accuracy of MRI for Identifying Radiographic High Slope (XR ≥ 12°). The blue solid line corresponds to the ROC curve, the grey dashed line indicates the line of no discrimination (AUC = 0.5), and the orange × marks the optimal cutoff value determined using Youden’s index.

Using XR ≥ 12° as the reference standard for defining a “high slope,” MRI showed excellent diagnostic performance, with an Area Under the Curve (AUC) of 0.841 (95% CI, 0.760–0.922), as illustrated in Figure 4. Based on Youden’s index, the optimal MRI cutoff (8.8°), sensitivity was 81.6% (95% CI, 65.7–92.3%), specificity 82.9% (95% CI, 72.0–90.8%), and accuracy 82.4% (95% CI, 73.9–89.1%). The positive predictive value was 72.1% (95% CI, 56.3–85.1%), and the negative predictive value was 89.2% (95% CI, 79.1–95.3%). These diagnostic metrics, along with the 2 × 2 classification results, are summarized in Table 2 and Table 3. Cohen’s κ reached 0.626 (95% CI, 0.469–0.775), indicating substantial agreement between modalities, and McNemar’s test confirmed the absence of systematic misclassification (*p* = 0.36). In comparison, applying an MRI threshold of 12° yielded markedly lower sensitivity (39.5%), despite high specificity (95.7%) and a lower Cohen’s κ (0.400).

### 3.5. Subgroup Analyses

No statistically significant differences in PTS measurements were found between males and females for either XR (mean difference −0.68°, *p* = 0.29) or MRI (mean difference −0.54°, *p* = 0.37). No significant correlations were found between PTS and age, BMI, or weight for either modality (all *p* > 0.07).

## 4. Discussion

The most important finding of this study is that radiographic measurements systematically overestimate MRI-based posterior tibial slope (PTS), preventing the two modalities from being used interchangeably in pediatric patients with ACL injury.

The present study also confirms that, although XR and MRI measurements are significantly correlated (*r* = 0.602; ρ = 0.604; *p* < 0.001), their agreement remains limited, with only moderate concordance between modalities.

The mean tibial slope measured on XR in our pediatric cohort with ACL injuries is consistent with values reported in the literature for similar populations. Vyas et al. [11] reported a mean medial PTS of 12.1 ± 3.3° in ACL-injured subjects, while O’Malley et al. [16] found a mean posterior tibial slope of 10.6 ± 3.1° in the ACL-injured group. Regarding MRI-based measurements, few studies have investigated medial PTS in pediatric patients, and the reported values remain inconsistent. Cooper et al. [20] reported a mean medial PTS of 5° in the ACL-injured group.

Previous pediatric studies have highlighted the relevance of tibial slope in ACL injury, with XR-based work by Vyas et al. [11] and MRI-based analyses by Dare et al. [12] reporting steeper slopes in injured children. However, none examined how XR and MRI measurements relate to each other. A recent adult-based study reported poor limits of agreement for MRI measurement techniques, underscoring methodological variability across modalities [21]. Importantly, no previous work has attempted to calibrate MRI values to established radiographic thresholds in children. The present study addresses this gap by quantifying cross-modal agreement and proposing an MRI cutoff corresponding to the conventional ≥12° radiographic definition.

In our study, tibial slope measurements across XR and MRI were significantly correlated (r = 0.6). In the literature, only a few studies in adult populations have explored this relationship, and their findings are discordant. Utzschneider et al. [7] reported a strong correlation between XR and MRI, with a Pearson correlation coefficient of *r* > 0.9 (*p* < 0.001), whereas Naendrup et al. [8] found a moderate correlation (*r* = 0.47). To our knowledge, no previous study has examined this correlation in a pediatric population.

Radiographs consistently yielded higher values than MRI, with an average difference of 1.8°. This finding is consistent with previous investigations in adult populations. Gwinner et al. [22] reported that PTS measured on MRI was significantly lower than on radiographs (4.2° ± 2.9° vs. 9.1° ± 3.6°; *p* < 0.0001). Similarly, Hudek et al. [19] found a significantly greater medial PTS on lateral radiographs (8.2°) compared with MRI (4.8°), with a mean difference of 3.4° (*p* < 0.0001).

Another important finding of the present study is that PTS measurements show a high degree of variability between the two methods, with individual discrepancies reaching approximately ±5°. In adults, Naendrup et al. [8] reported similar findings, with differences of up to 5° between radiographic and MRI-based measurements and standard deviations of up to 3° across different measurement techniques. Similarly, Amerinatanzi et al. [23] found significant differences in mean values between measurement methods (*p* < 0.005).

Because the 95% limits of agreement reached approximately ±5°, which exceeds the mean systematic difference between modalities, individual-level translation between XR and MRI is unreliable. Consequently, the proposed MRI threshold should be interpreted as a probabilistic approximation rather than a strict numeric equivalence.

These findings indicate that the two measurement methods cannot be used interchangeably at the individual level. Radiographic thresholds cannot be directly applied to MRI without a risk of misclassification. This observation is consistent with results reported in adult cohorts, such as those of Naendrup et al. [8], and further supports the need to validate tibial slope measurement methods specifically for pediatric populations.

In this study, an MRI cutoff of approximately 9° corresponded to the established radiographic pathological threshold of 12° reported in the literature [5], providing balanced sensitivity and specificity with substantial agreement between modalities. Adopting a lower cutoff therefore appears appropriate to avoid misclassification. However, it is important to note that this threshold has been defined in adults to characterize a “high slope” and is typically discussed in the context of anterior closing-wedge high tibial osteotomy during ACL reconstruction for graft failure. [24,25,26] Moreover, no standardized cutoff on cross-sectional imaging has been established for adults. Hudek et al. [19] suggested that direct conversion between MRI- and radiograph-based PTS measurements may be possible, whereas Naendrup et al. [8] warned that such conversion carries a risk of inaccuracy and should be avoided.

XR was treated as an anchor in the ROC analysis because the ≥12° threshold originates from adult radiographic literature and remains the conventional clinical benchmark. This does not imply methodological superiority of radiographs; it simply reflects current clinical practice and provides a historically established reference for comparison.

In pediatric populations, no pathological threshold has been defined on either XR or MRI. This raises the question of the clinical relevance and indications of corrective surgery, as well as its benefit–risk balance. The present study is clinically relevant, as understanding the relationship between radiographic and MRI measurements may improve clinical decision-making by enabling more accurate interpretation of tibial slope values across imaging modalities.

This study has limitations, including its retrospective, single-center design. Only the medial tibial slope was assessed, disregarding potential medial–lateral asymmetry; this choice reflected the superimposition of the medial plateau on lateral radiographs. In addition, the inter-modality ICC showed a wide confidence interval (0.186–0.696), ranging from poor to moderate agreement, indicating imprecision likely related to sample size and suggesting that the study may be underpowered to estimate inter-modality reliability with high precision. Inter-observer reliability could not be assessed because measurements were not duplicated by multiple reviewers, representing an additional methodological limitation. Separate Bland–Altman plots for inter-observer agreement were considered but not included because the study’s primary aim concerned inter-modality comparison rather than inter-observer variability. However, ICC values are provided to quantify reviewer consistency. The absence of a non-ACL-injured control group limits the generalizability of these findings to broader pediatric populations.

Future studies should include multicenter, prospective cohorts to validate these findings and to define standardized, clinically meaningful thresholds for pathological slope in children.

## 5. Conclusions

Radiographs and MRI scans show a strong correlation but systematically different measurements of posterior tibial slope in pediatric patients with ACL injuries. Radiographs yield higher values, with a mean bias of 1.8°, and the radiographic threshold for a high slope (≥12°) should not be directly applied to MRI. An MRI cutoff of approximately 9° may serve as a pragmatic reference for the interpretation of MRI-based measurements, requiring further validation.

## Figures and Tables

**Table 1:** Baseline characteristics of the study cohort.

Characteristic	Value
N pairs	108
Male, *n* (%)	74 (68.5)
Female, *n* (%)	34 (31.5)
Age (years),	13.9 ± 2.0
Weight (kg),	58.0 ± 14.1
BMI (kg/m^2^)	22.1 ± 4.1
XR PTS (°)	10.2 ± 3.1
MRI PTS (°)	8.4 ± 2.8

Values are presented as mean ± standard deviation unless otherwise specified.

**Table 2:** Diagnostic Performance at Optimal MRI Cutoff (8.8°) with XR ≥ 12° as Reference.

Metric	Value
AUC (95% CI)	0.841 (0.760–0.922)
Best MRI cutoff (°)	8.8
True Positive	31
False Positive	12
False Negative	7
True Negative	58
Sensitivity	81.6%
Specificity	82.9%
Positive Predictive Value	72.1%
Negative Predictive Value	89.2%
Accuracy	82.4%

**Table 3:** Diagnostic Cross-Tabulation of MRI 8.8° Cutoff Using XR ≥ 12° as Reference Standard.

	XR ≥ 12°	XR < 12°	Total
MRI ≥ 8.8°	31	12	43
MRI < 8.8°	7	58	65
Total	38	70	108

## Data Availability

The data underlying this study are not publicly available due to ethical and privacy restrictions related to pediatric patient information. Anonymized data may be made available from the corresponding author upon reasonable request.

## Supplementary Materials

The following supporting information can be downloaded at: https://www.mdpi.com/article/10.3390/jcm15010064/s1, File S1: The GRRAS (Guidelines for Reporting Reliability and Agreement Studies) checklist.

## Abbreviations

The following abbreviations are used in this manuscript:

ACL	Anterior Cruciate Ligament
ACLR	Anterior Cruciate Ligament Reconstruction
AUC	Area Under the Curve
BMI	Body Mass Index
CI	Confidence Interval
CT	Computed Tomography
ICC	Intraclass Correlation Coefficient
MPTS	Medial Posterior Tibial Slope
MRI	Magnetic Resonance Imaging
MRI-LA	MRI Longitudinal Axis
NPV	Negative Predictive Value
PACS	Picture Archiving and Communication System
PPV	Positive Predictive Value
PTS	Posterior Tibial Slope
ROC	Receiver Operating Characteristic
SD	Standard Deviation
TP/FP/TN/FN	True Positive, False Positive, True Negative, False Negative
XR	X-Ray
