# Supplementary material for: Correlation Between Radiographic and MRI Posterior Tibial Slope Measurement on a Pediatric Population
Source: J Clin Med. 2025 Dec 22;15(1):64. doi: 10.3390/jcm15010064 (PMC12786420; doi:10.3390/jcm15010064)
Supplement: Supplementary file 1 [file jcm-15-00064-s001.zip › jcm-4032968-supplementary.pdf]

GRRAS (Guidelines for Reporting Reliability and Agreement Studies) Checklist

Item to be reported		Reported on Page Number/Line Number	Reported on Section/Paragraph
**TITLE AND ABSTRACT**			
1	Identify in title or abstract that interrater/intrarater reliability or agreement was investigated.	Page 1 / Lines 18 – 25,	
**INTRODUCTION**			
2	Name and describe the diagnostic or measurement device of interest explicitly.	Page 2 / Lines 49 – 56	
3	Specify the subject population of interest.	Page 2 / Lines 57 – 62	
4	Specify the rater population of interest (if applicable).	Page 4 / Lines 115 – 116	
5	Describe what is already known about reliability and agreement and provide a rationale for the study (if applicable).	Page 2 / Lines 53 – 56,	
**METHODS**			
6	Explain how the sample size was chosen. State the determined number of raters, subjects/objects, and replicate observations.	Page 3 / Lines 76 – 81	
7	Describe the sampling method.	Page 3 / Lines 76 – 81	
8	Describe the measurement/rating process (e.g. time interval between repeated measurements, availability of clinical information, blinding).	Page 3 / Lines 85 – 94	
9	State whether measurements/ratings were conducted independently.	Page 4 / Lines 115 – 116	
10	Describe the statistical analysis.	Page 4 / Lines 119 – 136	
**RESULTS**			
11	State the actual number of raters and subjects/objects which were included and the number of replicate observations which were conducted.	Page 5 / Lines 141 – 145	
12	Describe the sample characteristics of raters and subjects (e.g. training, experience).	Page 5 / Lines 142 – 145	
13	Report estimates of reliability and agreement including measures of statistical uncertainty.	Page 5 / Lines 150 – 154	
**DISCUSSION**			
14	Discuss the practical relevance of results.	Page 8 / Lines 195 – 198	

**AUXILIARY MATERIAL**			
15	Provide detailed results if possible (e.g. online)		

Article information: https://dx.doi.org/10.21037/qims-21-1205
*As the checklist was provided upon initial submission, the page number/line number reported may be changed due to copyediting and may not be referable in the published version. In this case, the section/paragraph may be used as an alternative reference.
